# Does Publication in Top-Tier Journals Affect Reviewer Behavior?

**DOI:** 10.1371/journal.pone.0006283

**Published:** 2009-07-21

**Authors:** Lonnie W. Aarssen, Christopher J. Lortie, Amber E. Budden, Julia Koricheva, Roosa Leimu, Tom Tregenza

**Affiliations:** 1 Department of Biology, Queen's University, Kingston, Ontario, Canada; 2 Department of Biology, York University, Toronto, Ontario, Canada; 3 National Center for Ecological Analysis and Synthesis (NCEAS), Santa Barbara, California, United States of America; 4 School of Biological Sciences, Royal Holloway University of London, Egham, Surrey, United Kingdom; 5 Department of Plant Sciences, University of Oxford, Oxford, United Kingdom; 6 Centre for Ecology and Conservation, School of Biosciences, University of Exeter, Cornwall Campus, Penryn, United Kingdom; Stockholm University, Sweden

## Abstract

We show that when ecologists act as reviewers their reported rejection rates recommended for manuscripts increases with their publication frequency in high impact factor journals. Rejection rate however does not relate to reviewer age. These results indicate that the likelihood of getting a paper accepted for publication may depend upon factors in addition to scientific merit. Multiple reviewer selection for a given manuscript therefore should consider not only appropriate expertise, but also reviewers that have variable publication experience with a range of different journals to ensure balanced treatment. Interestingly since age did not relate to rejection rates, more senior scientists are not necessarily more jaded in reviewing practices.

## Introduction

In the peer-review of publications, scientists alternate between two roles: one as author and the other as reviewer [1]. An important but largely unexplored issue is the extent that experience as an author affects his/her behavior as a reviewer and vice versa. It is generally assumed that experience in science enhances our capacity as scientists, however it is also possible that it introduces an element of bias in expectations as our own experiences change. Unfortunately, peer-review is not a perfectly objective system of assigning merit [2] and few would argue that this is the case. Nonetheless, we rely on it as a means to both filter research into appropriate venues and assess whether the research is valid, useful, novel, and repeatable. In any given instance, it would be desirable to ensure that the panel of reviewers selected to vet the research is representative of the specific subdiscipline and fair, i.e. to an extent detached from the potential success of the authors. The former instance is generally untested and likely varies by editor preference while the latter instance is likely just assumed.

If objective assessment of potential publication by others is one of our principal activities, then the effect of experience as referees needs critical examination, particularly since assessment could be balanced by selection of different categories of referees if they exist. In several instances, it has been shown that ecologists who publish more papers experience higher rejection rates of their manuscripts [3], [4]. Here we ask: when ecologists change hats and act as reviewers, do they also vary in predictable ways in the rejection rates that they recommend? We explore whether two criteria likely used frequently by editors – publication success of the reviewer (is this individual a successful expert in the field?) and scientific age (is this individual experienced within the field?) – relate to the reported rejection rates recommended by reviewers.

## Methods

As part of an NCEAS working group on publication bias, we conducted an online survey of ecologists to develop a profile of their experiences with publication and review. A total of 17 open-ended, multiple choice, and likert-scale questions relating to the publication process were included in the survey, however only those pertaining to this paper were analysed and reported here (Appendix S1). Specifically, we asked respondents to indicate the proportion of the manuscripts that they reject as reviewers: 0–25%, 26–50%, 51–75%, or 76–100% (Q3, Appendix S1), which (if any) of the listed high-impact factor journals they had published in (Q1, Appendix S1) and the year of their first peer-reviewed publication (Q4, Appendix S1). The group of high impact factor journals publishing ecology and evolutionary biology articles were selected based on their 2004 impact factor. Nature, Science, PNAS and Current Biology were also included, as they are top-tier biology journals even though not listed by ISI as ecology. We excluded those journals focusing on reviews (e.g. TREE, Annual Review of Ecology, Evolution and Systematics) and specialty journals (e.g. Molecular Ecology, Global Change Biology). Despite only recent circulation, we included PLoS Biology which began in 2003 but was already receiving high citations. The final list (‘top-ten’) comprised Nature, Science, Current Biology, PNAS, Ecological Monographs, American Naturalist, Ecology, Ecology Letters, Evolution and PLoS Biology. We assigned a ‘rejection intensity index’ of 1, 2, 3 or 4, to the categorical proportion estimates of rejection rate and subtracted the year of first publication from the survey date to obtain the number of years since first publication, a surrogate for scientific age.

The survey was posted online from May 4th, 2006 to November 4th, 2006 and was distributed to the Ecological Society of America (ECOLOG) and EvolDir mailing lists as well as promoted at international ecological and evolutionary conferences and posted on the working group website. These distribution lists were selected as a representative means to target ecologists and evolutionary biologists. The extent to which individual respondents subscribe to both list-serves was unknown hence the minimum (assuming there was complete overlap in subscribers to both list-serves) and maximum (where there was no subscription overlap) population sizes ranged from 6000 to 12 200. After removal of duplicates and incomplete surveys, the sample size was 1334 responses, representing between 11% and 22% of the total population solicited. We were unable to test for response bias as non-respondents could not be tracked due to the use of list-serves for survey distribution [5]. It was also not possible to accurately verify the responses using ISI since multiple authors across disciplines can have the same name and ISI does not cover all peer-reviewed journals. However, while rejection rates are perceived estimates, we believe the error associated with recall of first publication year and publication in top journals to be low given the importance of publication history and associated metrics in the assessment of scientific merit [6].

Chi-square tests were used to compare the distribution of rejection rate categories among respondents with and without publication experience in high impact factor journals. We calculated Spearman rank correlation coefficients (*r*_s_) to examine the association between the recommended rejection rates and reviewer's scientific age and the number of high impact factor journals in which he/she has published. Sensitivity analyses via exclusions were also used to ensure that a relationship detected was not a product of single individuals.

## Results

**Figure 1:**
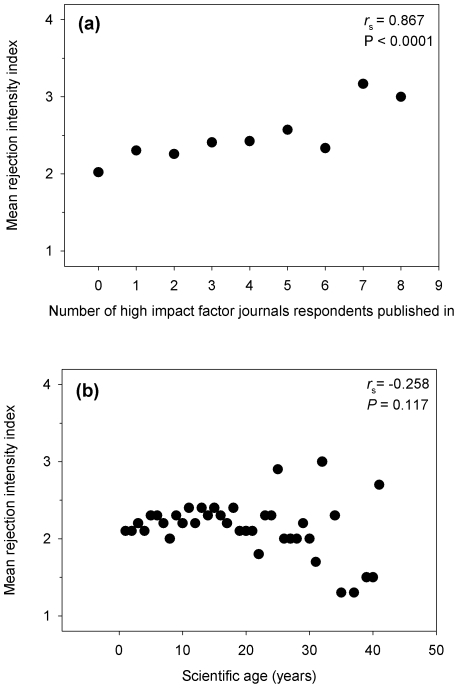
Results from a ‘publishing and reviewing’ survey of ecologists showing the relationship between mean manuscript rejection rate as a reviewer (mean rejection intensity index) and: (a) the number of high impact factor journals (out of a selection of ‘top-ten’) that respondents had previously published in (N = 1101); and (b) the mean ‘scientific age’ of respondents (N = 1235). Respondents were assigned a ‘rejection intensity index’ of 1, 2, 3, or 4 based on whether they stated a recommended rejection rate of 0–25%, 26–50%, 51–75%, or 76–100% respectively. ‘Scientific age’ is defined as the number of years since a respondent's first peer-reviewed publication. Increasing variance at higher scientific ages is due to smaller sample sizes. Spearman rank correlation coefficients (*r*_s_) and associated *P*-values are shown for plotted data.

Respondents reported significantly different recommended rejection rates of reviewed manuscripts depending on publication experience in high-impact-factor (IF) journals (Table 1). Furthermore, respondents that publish in a greater number of high-IF journals generally recommend rejection of a higher proportion of the manuscripts reviewed (Fig. 1a). There was no relationship between an individual's manuscript rejection rate and scientific age (inferred by the number of years since first peer-reviewed publication) (Fig. 1b). However, there is a positive relationship between scientific age and number of reviews conducted per year [7]. Rejection rate is also higher for those who review more papers per year (Spearman rank correlation = 0.201, P<0.001), but the number of reviews per year (5) did not differ between reviewers that had published within the top-ten journals (n = 729) versus those that had not (n = 603) (Mann Whitney U = 772738.5, P = 0.349).

**Table 1:** A 2×4 contingency table of number of respondents from a ‘publishing and reviewing’ survey for ecologists based on whether they stated a recommended rejection rate (as a reviewer) of 0–25%, 26–50%, 51–75%, or 76–100%, versus whether or not they had previously published in at least one high-IF journal (out of a selection of ‘top-ten’) [χ^2^ = 35.7; *P*<0.0001].

		WITHOUT previous publication in a high-impact-factor journal	WITH previous publication in a high-impact-factor journal	Total
Recommended rejection rate as a reviewer (percentage of papers reviewed)	0–25%	139	121	260
	26–50%	185	265	450
	51–75%	88	201	289
	76–100%	30	72	102
	Total	442	659	1101

## Discussion

Peer review is a necessary tool in science. It improves science and gives our work credibility. However, reviewer assignment is not a random draw nor without consequence; the selection of a given reviewer can affect the fate of a paper and the importance of referee selection has been critically overlooked [8]. Here, we examined whether referees with different publication track records report different recommended rejection rates when reviewing. Our results show that ecologists do not become more critical reviewers over time *per se*, but that they do likely become more critical as they publish more in high-IF journals. The increase in recommended rejection rates can be as much as double or triple (i.e. from 26–50% to 51–75%, Fig. 1a). Arguably, those who publish in more high-IF journals may also do more reviewing for these same journals [7], which may have a tradition of demanding relatively high overall rejection rates [9]. Nevertheless, these journals are also likely to receive relatively high quality submissions, and these reviewers are also likely to be doing much or most of their reviewing for journals with low to intermediate impact factors, given that these journals vastly outnumber high-IF journals. Those who publish in more high-IF journals may also have more experience with rejection from these journals, which might also affect their assessment of publication merit generally. Publication in (and/or rejection from) top-tier journals may not be causing these referees to be more negative but they may hold different expectations or value criteria for merit differently. For instance, novelty is arguably a key element required for publication in top-tier journals [10], yet for other journals alternative criteria may be differentially weighted such as empirical rigor, or repeatability.

The expectations we use to assign merit to the research we review is based on our understanding of the field, what we view as novel, needed, or appropriate, but also on which journals we read more often, and where we publish. We cannot assess whether reviewers with more publication experience in high-IF journals are unduly critical (i.e. reject manuscripts that actually merit publication), or whether those with less experience are insufficiently critical (i.e. fail to notice significant shortcomings). Nevertheless, variation in rejection rate by reviewer attributes, as reported here, represents a potential reviewer bias, which is likely to affect community-level perception and assignment of relative merit in the peer-review process [2]. Our data suggest that several formal elements could be included in the peer review process to ensure that at the very minimum the panel of peers is representative of that specific community of scientists. Scientific age apparently has no influence, but our results suggest that selection of referees should be balanced on a per manuscript basis by publication record in the top-tier journals. In addition, based on our results, many submitting authors might be persuaded to generally avoid suggesting names for reviewers that have published extensively in high-IF journals.

## Supporting Information

Appendix S1 (0.03 MB DOC)
